# CHD4 orchestrates the symphony of T and B lymphocytes development and a good mediator in preventing from autoimmune disease

**DOI:** 10.1002/iid3.644

**Published:** 2022-06-06

**Authors:** Miaomiao Jia, Xueqin Zou, Shuying Yin, Weihong Tian, Yangjing Zhao, Hui Wang, Guoying Xu, Weili Cai, Qixiang Shao

**Affiliations:** ^1^ Reproductive Sciences Institute Jiangsu University Zhenjiang Jiangsu P.R. China; ^2^ Department of Immunology, School of Medicine Jiangsu University Zhenjiang Jiangsu P.R. China; ^3^ School of Medical Science and Laboratory Medicine, Institute of Medical Genetics and Reproductive Immunity Jiangsu College of Nursing Huai'an Jiangsu P.R. China

**Keywords:** autoimmune disease, B cells, chromodomain helicase DNA binding protein 4, nucleosome remodeling and deacetylation complex, T cells

## Abstract

Chromodomain helicase DNA binding protein 4 (CHD4) is an ATPase subunit of the nucleosome remodeling and deacetylation complex. It has been implicated in gene transcription, DNA damage repair, maintenance of genome stability, and chromatin assembly. Meanwhile, it is highly related to cell cycle progression and the proceeding of malignancy. Most of the previous studies were focused on the function of CHD4 with tumor cells, cancer stem cells, and cancer cells multidrug resistance. Recently, some researchers have explored the CHD4 functions on the development and differentiation of adaptive immune cells, such as T and B lymphocytes. In this review, we will discuss details of CHD4 in lymphocyte differentiation and development, as well as the critical role of CHD4 in the pathogenesis of the autoimmune disease.

## Graphical abstract

Chromodomain helicase DNA binding protein 4 (CHD4) is an ATPase subunit of the nucleosome remodeling and deacetylation complex. It has been implicated in gene transcription, DNA damage repair, maintenance of genome stability, and chromatin assembly. Recently, some researchers have explored the CHD4 functions on the development and differentiation of adaptive immune cells, such as T and B lymphocytes. In this review, we will discuss the details of CHD4 in lymphocyte differentiation and development, as well as the critical role of CHD4 in the pathogenesis of the autoimmune disease.

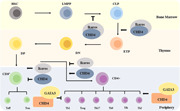

## 1 INTRODUCTION

Although many approaches have been used to investigate the origin of autoimmune diseases, the understanding of the pathogenic mechanisms remains poorly defined. Encouragingly, plenty of studies have found that the epigenetic changes came earlier than autoimmune diseases and epigenetic processes were involved in the regulation and guidance of lymphocytes development and differentiation. More critically epigenetic processes also affect autoimmune diseases occurrence, development, relapse, and drug resistance. The developmental diversity of T cell receptor (TCR) and B cell receptor (BCR) is an indispensable critical stage in lymphocytes development. TCR and BCR gene clusters have a similar structure and analogous process of rearrangement. During this process, T and B cells differentiate into mature lymphocytes and respond to antigen‐specific stimulation via functional BCR or TCR, and eventually differentiate into plasma cells or sensitized T cells.^1^, ^2^

Previous studies have revealed that RAG1/2, a recombinase, interacted with several transcription factors, such as RUNX, E2A, GATA‐3, and CTCF to govern the promoters and/or enhancers of TCR gene clusters. Meanwhile, early B cell factor 1 (EBF1), B lymphocyte‐induced maturation protein 1 (Blimp‐1), B‐cell lymphoma 6 protein (Bcl‐6), and histone deacetylases (HDACs) regulate B‐cell development, differentiation, and somatic hypermutation/class switch recombination (CSR).^1^ These tentative factors have been found relating to lymphocytes development and autoimmune diseases.^3^, ^4^ However, how chromosome remodeling is involved in autoimmune diseases remains largely unknown. Chromodomain helicaseDNA binding protein 4 (CHD4), a newly identified transcription factor, contributes to transcriptional repression, chromatin organization, genomic stability, and cell development.^5^ Emerging data indicated the relevance of CHD4 in T and B lymphocytes development and gene rearrangement that attributed to various cancers and several autoimmune diseases.^6^ Here, we reviewed the current knowledge of CHD4 and mainly focused on its function on lymphocytes differentiation and development as well as its role in autoimmune diseases.

## 2 CHD4

### 2.1 The discovery and structure of CHD4

**Figure 1:**
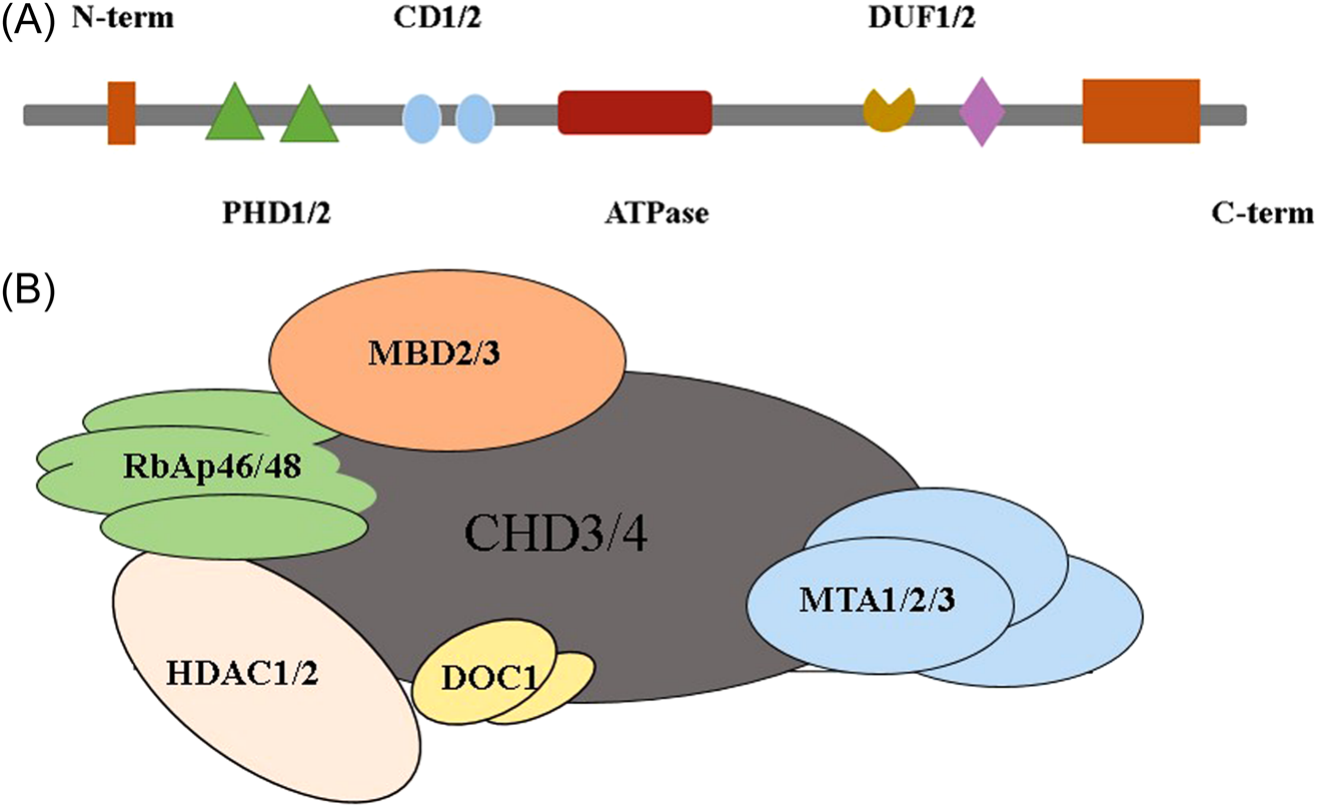
Structure of CHD4 and nucleosome remodeling and deacetylation (NuRD) complex. (A) Domain structure of CHD4. CHD4, a major component of NuRD also called Mi‐2β, is an indispensable part of the CHD family. CHD4 has two plant homeodomain fingers, which can directly connect to H3K9me3 (an epigenetic modification to the DNA packaging protein Histone H3), a chromodomain, a core of ATPase/helicase module, and two unknown domains. (B) Structure of CHD4 and NuRD complex. NuRD contains six unique subunits: histone deacetylases (HDAC1/2), ATP‐dependent remodeling enzymes (CHD3/4), histone chaperones (RbAp46/48), CpG‐binding proteins (MBD2/3), specific DNA‐binding proteins (MTA1/2/3), and DOC‐1. HDAC1/2 attaches great importance to transcriptional regulation in eukaryotic cells. RbAp46 and RbAp48 are WD40‐repeat histone chaperones and chromatin adapters that reside in multiple complexes involved in the maintenance of chromatin structure. MBD3, a methyl‐CpG‐binding domain (MBD)‐containing protein, recruits the complex to methylated DNA. The role of MTA1/2/3 has not been fully identified at all. DOC‐1 also called p12, is a growth suppressor, isolated from normal keratinocytes.

CHD4, an ATP‐dependent remodeling enzyme, is also called Mi‐2β. It is located on chromosome 12 in humans and 6 in mice. CHD4 encodes a 260 kDa protein, which is widely expressed in animals and plants, except in yeast; meanwhile, it contains a chromodomain, a core of ATPase/helicase module, two plant homeodomain (PHD) fingers that directly connect to H3K9me3, and two unknown domains. The chromo‐domain is a chromatin‐binding domain and essential for proper ATP‐dependent nucleosome remodeling activity. PHD fingers do not enhance the activity of ATPase, in turn, they augment interaction between the PHD fingers and chromo‐domain (CD) (Figure 1A).^7^, ^8^ CHD4 was firstly proposed as an autoantigen in autoimmune disease dermatomyositis (DM), which was a subtype of inflammatory myopathy.^9^ Later on, DM patients were found with a high level of anti‐Mi‐2β/CHD4 in the serum.^5^, ^10^, ^11^

### 2.2 The biological function of CHD4

CHD4 is involved in multiple nuclear processes, including DNA damage repair (DDR) and cell cycle transition.^5^, ^12^, ^13^ It is associated with tumor suppressor genes, noncoding RNAs, tumor movement, and drugs resistance in cancer therapy.^14^, ^15^, ^16^, ^17^ Later on, increasingly works have demonstrated that CHD4 is closely related to the development and differentiation of lymphocytes and immune system disease.^10^, ^18^, ^19^

## 3 CHD4 DOMINATES LYMPHOCYTE DEVELOPMENT AND DIFFERENTIATION

### 3.1 CHD4 in lymphocyte development

CHD4 acts as a core part of the nucleosome remodeling and deacetylation (NuRD) complex and belongs to the class Ⅱ subfamily of CHD ATPase. NuRD, also known as Mi‐2, is a complex containing both HDAC and ATP‐dependent chromatin‐remodeling functions, which is similar to the switching defective/sucrose nonfermenting complexes from the *Saccharomyces cerevisiae*. The complex consists of six unique subunits, including histone deacetylases 1/2 (HDAC1/2), CHD3/4, histone chaperones (retinoblastoma protein‐associated protein 46/48, RbAp46/48), CpG‐binding proteins (methyl‐DNA binding protein 2/3, MBD2/3), specific DNA‐binding proteins (metastasis‐associated proteins1/2/3, MTA1/2/3), and deleted‐in‐oral‐cancer‐1 (DOC‐1).^7^, ^13^ The structure and specific biological functions are shown in Figure 1B and Table 1.

**Table 1:** Components involved in NuRD

Components	Function	References
Histone deacetylases (HDAC1/2)	Interacts with inhibiting factors and plays a critical role in transcriptional regulation in eukaryotic cells	[43]
ATP‐dependent remodeling enzymes (CHD3/4)	Relates to differentiation of B and T lymphocytes, cell cycle fate, and DNA damage or repair	[5,10]
Histone chaperones (RbAp46/48)	Involves in the maintenance of chromatin structure	[44]
CpG‐binding proteins (MBD2/3)	Recognizes both methylated CG (mcg)‐and hydroxymethylated CG (hmcg)‐containing DNA	[45]
Metastasis associated proteins1/2/3(MTA1/2/3)	Overexpressed in hypoxia. Enhances the expression of HIF‐1. Linked to the estrogen receptor (ER) in cancer and mammary gland development	[13]
Deleted‐in‐oral‐cancer‐1 (DOC‐1)	A growth suppressor that has been identified and isolated from normal keratinocytes	[46]

Recent studies have shown that CHD4 played an essential part in the process of lymphocytes generation and differentiation^10^ as well as autoimmune diseases.^19^

#### 3.1.1 CHD4 in T cell development

T lymphocytes originate from bone marrow (BM) progenitors that migrate to the thymus and undergo a selection of maturation, and subsequently, export to the periphery, stimulated by antigen and initiate an immune response.

**Figure 2:**
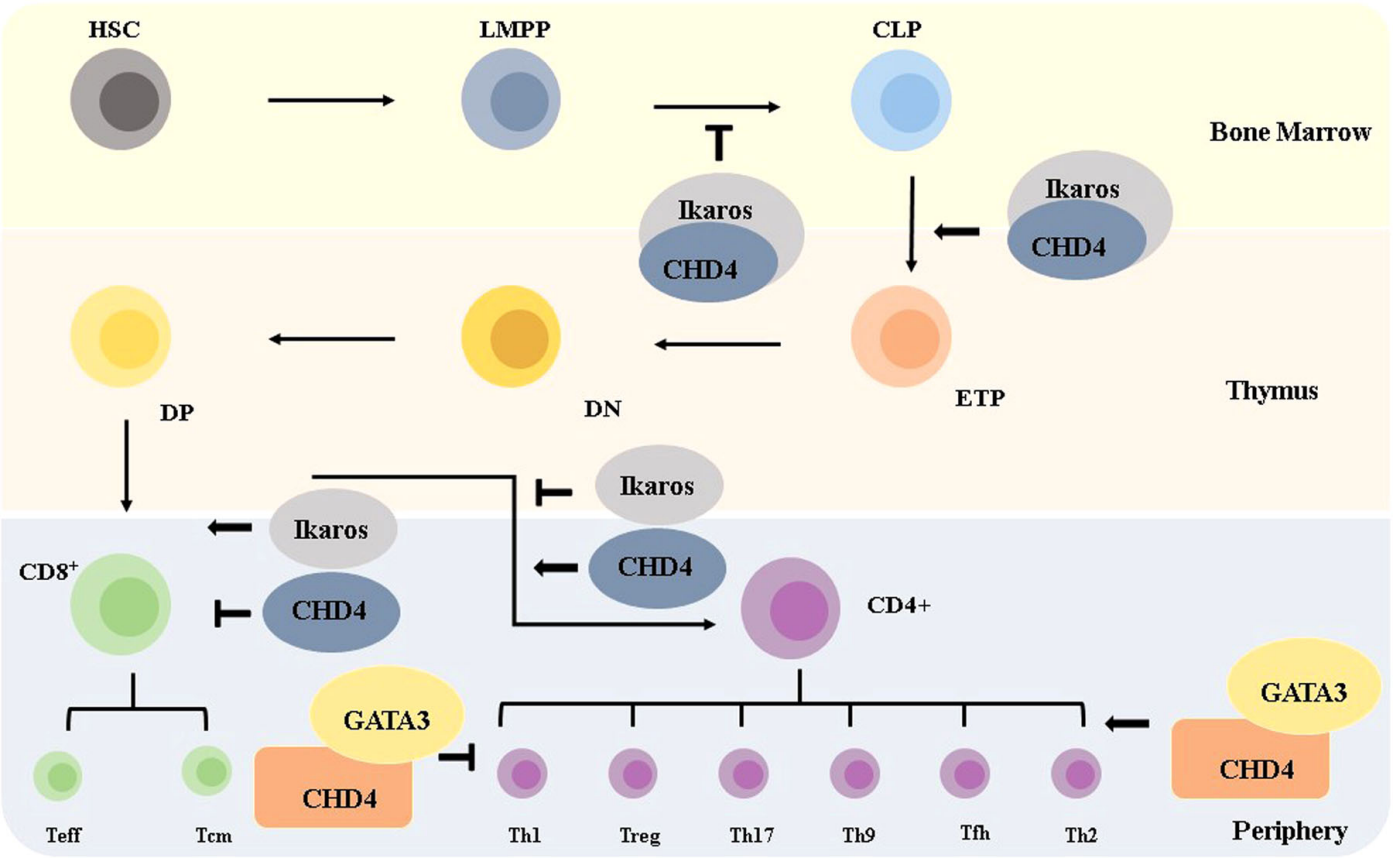
CHD4 and T cell development. T lymphocytes originate from bone marrow progenitors, which migrate to the thymus, undergo positive and negative selection for maturation, and subsequent export to the peripheral lymphoid organs. HSC, hematopoietic stem cells; LMPP, lymphoid primed multipotent progenitors; CLP, common lymphoid progenitors; ETPs, early thymic progenitors; CD4^+^ and CD8^+^ mean single positive (SP) T cells; Th cells, T helper cells; Tfh, follicular T cell; CD8^+^ effector T cells (Teff), and CD8^+^central memory T cells (Tcm).

The differentiation of HSCs into lymphoid progenitor cells in BM is regulated by CHD4 and Ikaros. Ikaros, a zinc‐finger protein, is crucial for lymphocytes development and is widely expressed in all stages of T cells.^20^ In previous reports, CHD4 has been demonstrated to interact with Ikaros to maintain early hematopoiesis and multilineage differentiation. The researchers found that hematopoietic stem cells were more likely to differentiate into myeloid and erythroid during differentiation in CHD4 and Ikaros double knockout mice. The isolated HSCs, lacking CHD4 and Ikaros, could not be induced into lymphoid progenitor cells in vitro.^21^, ^22^ These results suggested that CHD4 and Ikaros inhibited myeloid or erythroid differentiation and promoted lymphoid differentiation (Figure 2).^10^

The development of T cells in the thymus is a highly dynamic process. T cells first go through *Tcr* rearrangement, then undergo positive and negative selection.^2^, ^6^, ^23^ The progenitors of T cells migrate to the thymus microenvironment and undergo multiple stages to develop into mature T cells (Figure 2).

CHD4 and Ikaros are crucial to the recombination of TCRα and TCRβ genes. To test how CHD4 regulated V‐D‐J recombination at *TCRα* genes, an Ikaros‐deficient, single functional *TCRβ gene* rearrangement and expressing surface pre‐TCR CD4^‐^CD8^‐^ mouse thymoma cell line JE131 was used.^24^ Ikaros were reintroduced into JE131 cells via a retroviral vector and the ratio of α locus transcription and TCR *Vα* to *Jα* rearrangements was increased and the pre‐TCR DN thymocytes were matured to TCRαβ^+^ DP thymocytes. Moreover, the knockdown of CHD4 increased the Ikaros‐dependent V‐D‐J rearrangement, indicating that CHD4 could inhibit the V‐D‐J rearrangement of *TCRα* gene loci. In short, CHD4/Ikaros complex promoted pre‐T cells to αβTCR^+^ CD4^+^CD8^+^ thymocytes transition and controlled Vα/Jα recombination in T cells by influencing access of the transcription and recombination machinery to the TCR α loci (Figure 2).^10^, ^24^, ^25^ However, the mechanism of this aspect still needs to be further explored.

In the stage of positive selection, one study used CHD4 knockout mice to identify that loss of CHD4 led to a decrease in thymocyte subpopulations, including the decrease of DP and CD4^+^ thymocytes, but an increase in CD8^+^ SP thymocytes. The results suggested that CHD4 regulated the differentiation of thymocytes from DN to DP stage. ChIP analysis illustrated that CHD4 was recruited to the *Cd4* gene enhancers through its interaction with HEB (Tcf12) and p300. Hence, CHD4 is a positive factor in CD4^+^ T cell development. However, in CHD4 and Ikaros double knockout mice, it has been revealed that CHD4 and Ikaros proteins have antagonistic effects on T cells. Ikaros may upregulate *Cd8* gene expression by increasing chromatin accessibility to promote the development of CD8^+^ T cells (Figure 2).^10^, ^26^ After positive selection, thymocytes undergo negative selection. During this period, CHD4 has a critical function in central immune tolerance. It recognizes the promoter regions of Fezf2‐dependent genes and drives the expression of Aire‐induced self‐antigen genes via super‐enhancers to eliminate the autoreactive T cells and induces the central immune tolerance.^6^

#### 3.1.2 CHD4 in B cell development

**Figure 3:**
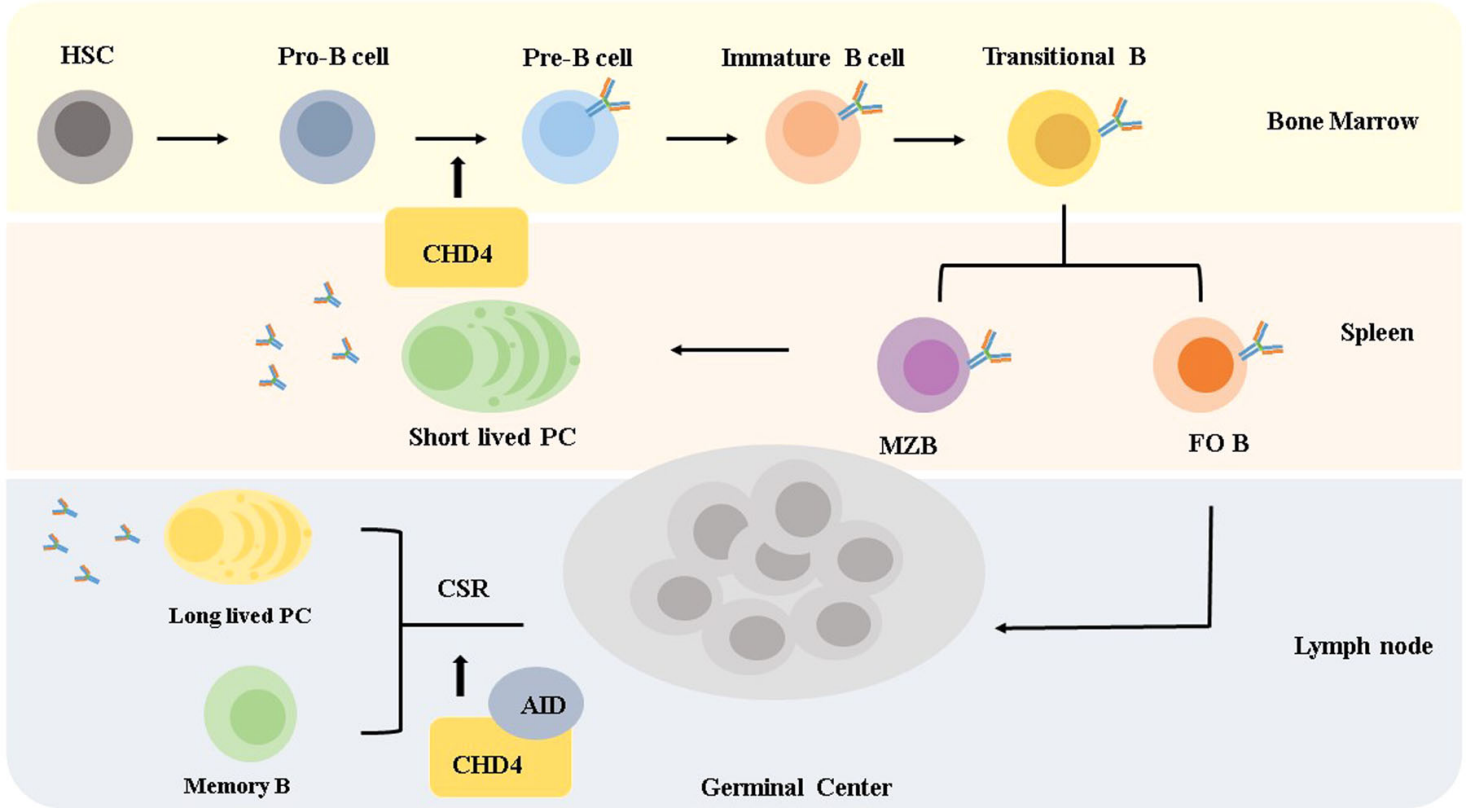
CHD4 and B cell development. B cells develop from hematopoietic stem cells in the bone marrow. FOB, follicular B cell; HSCs, hematopoietic stem cells; MZB, marginal zone B cell.

B lymphocytes develop from lymphoid stem cells in the BM and are primarily found in peripheral immune organs.^27^, ^28^ The development of B cells in BM go through the stages of pro‐B cells, pre‐B cells, immature B cells, and mature B cells. The central tolerance of B cells is generated by clonal deletion at the stage of immature B cells. (Figure 3).^28^

During B cell development, EBF1 is required for B lymphopoiesis, pre‐BCR function, Pax5 controls B cell lineage progression, and suppresses incorrect genes.^1^ The membrane expression of BCR V‐D‐J recombination of Ig genes in the BM is the most essential characteristic of immature B cells.^28^ Recent studies demonstrated that CHD4–NuRD complexes regulated chromatin accessibility in B cell‐specific transcription^28^, ^29^, ^30^ and CHD4 was recently shown to be essential for B cell development and V‐D‐J rearrangements at immunoglobulin heavy chain (*Igh)* loci.^31^

Considering there were still no clear demonstrations of how CHD4 contributes to B cell lineage‐specific progression, James R. Hagmana and his colleagues bred CHD4‐deficient mice.^31^ Their results suggested that B cells were arrested from pro‐B cells to the pre‐B cell stage (Figure 3). Simultaneously, IL‐7 stimulated B cells from CHD4‐deficient mice, confirming that IL‐7 signaling was defective in CHD4‐deficient mice. Moreover, in the process of B cell development, lacking CHD4 led to impairing of locus contraction and the efficiency of *Igh* rearrangements along with dysregulation of DDR pathways. However, it is doubtful whether the high frequency of DNA damage is due to the absence of CHD4, which blocks essential transcriptional programs to carry out the function.^31^ Interestingly, they also found that CHD4 deficiency mice had a sharply decreasing usage of distal gene segments, suggesting that CHD4 had a role in locus contraction of *Igh* loci, but further details of this mechanism remain to be determined.^8^ Together, CHD4 is important for establishing a pro‐B cell program and is highly associated with lineage‐appropriate transcriptional programs and V‐D‐J rearrangements at *Igh* loci.^31^

### 3.2 CHD4 in lymphocytes differentiation

#### 3.2.1 CHD4 in T cell differentiation

GATA3 expresses in many embryonic and adult tissues, including adrenal glands, kidneys, central nervous system, inner ear, hair follicles, skin, and breast tissue. It belongs to the GATA (GATA‐binding protein) family that is comprised of GATA1 to 6 in mammals.^32^ It contains one or two C2‐C2‐type zinc‐finger motifs that recognize the consensus DNA sequence WGATAR (where W denotes A or T and R denotes A or G). Importantly, GATA3 is expressed in immune cells, including mature T cells, natural killer (NK) cells, and CD1‐restricted NKT cells.^33^

In peripheral immune organs, during the differentiation process, naïve T cells differentiate into Th2 cells. It has been elucidated that the activated CHD4/GATA3 complex recruits p300 and binds to the specific site control region of Th2‐type cytokine genes, while the GATA3/CHD4 complex interacts with HDAC2 and forms inhibitory GATA3/CHD4‐NuRD complex to TBX21; the former induces Th2 cytokine gene activation, while the latter inhibits TBX21 gene, thereby inhibiting Th1 differentiation and interferon‐γ expression.^34^

#### 3.2.2 CHD4 was highly related to CSR

After the immature B cells in BM form central immune tolerance. They develop into mature B cells and emigrate to the peripheral lymphoid tissue where they are only activated by foreign antigens to exert the adaptive immune response. B cells are activated by BCR and CD40L signals and gain the ability to continue to proliferate and differentiate. With the help of cytokines produced by Th cells, activated B cells form germinal centers and undergo Ig affinity maturation and CSR. Finally, these B cells differentiate into plasma cells or memory B cells and perform the humoral immune function.^28^, ^35^ CSR is pivotal for producing high‐affinity antibodies that play different functions.

Till now, the mechanism of how dynamic landscape regulation promotes CSR is still unclear. Some researchers assumed that CHD4 could be a factor interacting with activation‐induced cytidine deaminase (AID) to accelerate CSR^36^ (Figure 3) Various types of animal models were established to clarify the mechanism. In *Mb1* ^*cre/wt*^ *Chd4* ^*fl/fl*^ mice, B cells were arrested at the stage from pro‐B cells to pre‐B cells. The B cell arrest was reversed in p53 knockout mice, suggesting that the V‐D‐J arrangement stimulated the checkpoint protein p53 activation. However, the number of those two stages B cells was not recovered to the normal. Moreover, in *Cd19* ^*cre/wt*^ *Chd4* ^*fl/fl*^ mice, the splenocytes were isolated and treated with LPS and IL‐4 or LPS. The results revealed that loss of CHD4 impaired CSR to IgG1or IgG3, while it did not affect plasma blast differentiation. Notably, CHD4 is a critical factor for B cell development and differentiation and also influences GC response as well as CSR from IgM to IgG1 or IgG3. Besides, to further pursue the mechanism of CSR, scientists utilized ChIP experiments to test whether CHD4 interacts with AID, the results clarified that AID and CHD4 formed a protein complex to facilitate IgG CSR. CHD4 was also linked to AID during CSR and exerted its effectiveness in B cell proliferation via a p53‐dependent pathway.^8^ Collectively, it has been illustrated that deficiency of CHD4 causes the cell arrest from pro‐B cells to pre‐B cells phase, and CHD4 triggers depression of cell adhesion and cell migration signaling factors via enhancing chromatin accessibility at poised enhancers; meanwhile, it is a positive regulator of CSR.^31^

In summary, CHD4 forms a complex with Ikaros that inhibits myeloid and erythroid differentiation and promotes lymphoid differentiation. It also drives CD4^+^ T cells differentiation and inhibits the V‐D‐J rearrangement of *TCRα* gene loci. In the process of peripheral T cells differentiation, CHD4/GATA3 complex interacts with HDAC2, boosts Th2 cells differentiation, and decreases Th1 cell differentiation. Meanwhile, it has also been illustrated that CHD4 promotes pro‐B cells to pre‐B cells phase and is a positive regulator of CSR. However, unfortunately, no further report has been found that CHD4 is involved in the central tolerance of B cells.

## 4 THE ROLE OF CHD4 IN AUTOIMMUNE DISEASES

Previous research has shown that high levels of anti‐Mi‐2 in the serum of people with DM.^37^ DM is a subtype of inflammatory myopathy with specific skin and muscle involvement. A growing corpus of data suggested that DM‐specific autoantibodies define subsets with unique phenotypes. The subtype of the NuRD protein complex of Mi‐2α and Mi‐2β has first been described in DM.^11^ Several researchers utilized machine learning algorithms to show that there were different gene expressions profile in various DM. For example, calcium/calmodulin‐dependent protein kinase type 1 G (CAMK1G), early growth response protein 4 (EGR4), and CXC motif chemokine ligand 8 (CXCL8), also known as interleukin 8 (IL‐8), were highly expressed in ankylosing spondylitis (AS) but not in DM or other types of myositis, while apolipoprotein A4 (APOA4) was only expressed in anti‐3‐hydroxy‐3‐methylglutaryl‐CoA reductase (HMGCR) myopathy, and mucosal vascular addressin cell adhesion molecule 1 (MADCAM1) was exclusively expressed in anti‐Mi2‐positive DM.^38^

In a clinical investigation, anti‐Mi2 antibodies were detected in a mild DM and severe DM, whereas anti‐SAE1 was only found in an amyopathic form.^18^ Another study confirmed the clinical phenotype of DM via anti‐Mi‐2 autoantibodies from 58 recruited DM patients, with the prevalence and severity of clinical features as well as assessed longitudinal anti‐Mi2 autoantibody titers. These findings suggested that the progression of the disease was more serious in Mi‐2 positive patients than in anti‐Mi‐2 negative patients. Moreover, the level of anti‐Mi‐2 autoantibodies was associated with the severity of the disease and normalized in patients entering remission.^39^

Autoimmune antibodies are increased in almost all autoimmune diseases. B cells depletion with rituximab therapy exerts a great effect on the treatment of autoimmune diseases.^40^ To determine the longitudinal trends in serum levels of four myositis‐associated autoantibodies: anti‐Jo‐1, TIF1‐γ, SRP, and Mi‐2 after B cells deletion treatment, researchers tested these auto‐antibody levels via myositis core‐set measures. It had been illustrated that anti‐Jo‐1, anti‐TIF1‐γ, and anti‐Mi‐2 levels were decreased in myositis subjects after B cells depletion, and correlated with disease activity. However, anti‐SRP levels were only associated with longitudinal muscle enzyme levels. The close association between anti‐Jo‐1 levels and clinical outcomes suggested that anti‐Jo‐1 autoantibodies might be a good biomarker of disease activity.^39^

Increased CHD4 expression has been found in nuclear extracts of CD8^+^ T cells from AS patients.^41^ Meanwhile, the expression levels of the RUNX family and IRF family have similar expression profiles as that of CHD4. Most encouragingly, CHD4 is associated with both AS and DM activity, suggesting that it might be a potential biomarker during the autoimmune diseases process. These results provided new insights into novel therapeutic targets for the management of AS.^41^

## 5 DISCUSSION

The NuRD is a multisubunit complex that participates in the processes of histone deacetylation, histone demethylation, and nucleosome mobilization. CHD4, a major component of NuRD, is highly conserved in eukaryotes and widely existed in almost all species. CHD4 is important for the development and differentiation of lymphocytes. In early T cell development, it interacts with Ikaros and recruits to the gene locus and promoters involved in lymphocytic development and proliferation. Lacking CHD4 leads to autoreactive T cells infiltration in the peripheral organs. Additionally, it interacts with GATA3 and promoted the differentiation of Th2 cells. Far more importantly, loss of CHD4 causes CSR impairing and B cells arresting during the pro‐B cells to pre‐B cells phase.

Till now, the mechanism of CHD4 function has not yet been elucidated. It is unclear whether the CHD4 acts as an independent factor or a complex in lymphocyte development. The structure of the whole NuRD complex needs to be further elucidated to address how these factors access the macromolecular complexes and determine the molecular basis of interprotein interactions. Although some studies have suggested that CHD4 is linked to AID and recruits proper genes to *Igh* locus, it is still not known whether they function through binding of H3K9me3 or other modifications, which needs further exploration.

Not least, CHD4 is closely related to DM, but there is a gap between CHD4 and other autoimmune diseases. The serum level of anti‐Mi‐2β is increased in DM patients; however, few studies have explored whether it is also elevated in SLE or RA patients. Recently, CHD4 has been found to increase in nuclear extracts of CD8^+^ T cells from AS patients. It is well known that regulatory T cells (Treg) and Th17 have great relevance to autoimmune diseases. However, up to now, there is still no investigation on how CHD4 controls the differentiation of Treg and Th17. As we know, major studies are mainly focused on the mechanism of CHD4 in T cell and B cell development, but it has been reported that MBD2, a component of NuRD, facilitates the DNA demethylation of the Foxp3 upstream enhancer, promotes Foxp3 expression, and upregulates the suppressive function of Treg.^42^ Thus, we speculate that CHD4, as a major part of NuRD, may play a synergistic role in promoting Treg differentiation. In future studies, the relationship between CHD4 and Treg/Th17 may provide a new viewpoint for clinical diagnosis and disease surveillance of autoimmune diseases. In conclusion, CHD4 provides us with a new insight into autoimmune diseases and is also a potential biomarker for disease diagnosis and an effective therapeutic target.

## AUTHOR CONTRIBUTIONS

Miaomiao Jia writes the original graft. Qixiang Shao, Hui Wang, Xueqin Zou, Weihong Tian, Hui Wang, Yangjing Zhao, and Guoying Xu give suggestions about the structure of the manuscript and the writing of molecular mechanisms of CHD4 and summarize relevant studies. Qixiang Shao, Hui Wang, and Weili Cai helped to organize and revise the manuscript. And Qixiang Shao and Hui Wang were the funding recipients. All authors contributed to the article and approved the submitted version.

## CONFLICTS OF INTEREST

The authors declare no conflicts of interest.

## Data Availability

All the information included in this manuscript is available upon request by contact with the corresponding author.
